# Obstructed Bochdalek hernia in an adult

**DOI:** 10.1093/omcr/omac138

**Published:** 2022-12-16

**Authors:** Salih Mohammed, Hatim El-Basheir

**Affiliations:** University of Galway, Galway, Ireland; Royal care International Hospital, Cardiothoracic Surgery, Khartoum, Sudan

## Abstract

Congenital diaphragmatic hernia is a common neonatal anomaly. Presentation beyond childhood, however, is rare. We report, here, the case of a woman in her 50s who presented with an acutely obstructed posterolateral diaphragmatic hernia. The initial physical exam and radiological results could have led to an erroneous diagnosis of pneumothorax. We wish to emphasize the importance of having a high index of suspicion for this condition when cases with similar gastro-respiratory symptoms are encountered.

## INTRODUCTION

Congenital diaphragmatic hernias (CDHs) are a common entity with an incidence of one in 3000 births. The late fusion of the oesophagus and the septum transversum during development underlies the vulnerability for the development of CDHs. Ninety percent of CDHs are reported to involve the posterolateral aspects of the diaphragm as described by Bochdalek in 1848. Bochdalek hernias (BHs) are very rare beyond childhood with an incidence of 0.17% and are often incidentally discovered [1]. Symptomatic hernias are misdiagnosed in up to 38% of cases leading to unnecessary interventions such as chest tube insertion or failure to diagnose which could lead to strangulation of the herniated content and death [2].

## CASE PRESENTATION

A woman in her 50s presented to our Emergency Department with a 5-day history of dull abdominal pain and vomiting which later started to be associated with streaks of blood. Two days before presentation she developed breathlessness which was aggravated by food intake and associated with intermittent episodes of dry cough and wheezes. She had no chronic conditions, had no surgery or trauma and was not taking any long-term medication. On examination, she was dyspneic and unable to lie flat. Her oxygen saturation was 94% on 4 L/min of oxygen. She had a respiratory rate of 30/min, a heart rate of 107/min and a blood pressure of 177/100 mmHg. She was afebrile. Chest physical examination revealed a hyper-resonant percussion note on the left side with decreased air entry and bilateral wheezes. Her abdomen was distended with a tympanic percussion note.

## INVESTIGATIONS

**Figure 1:**
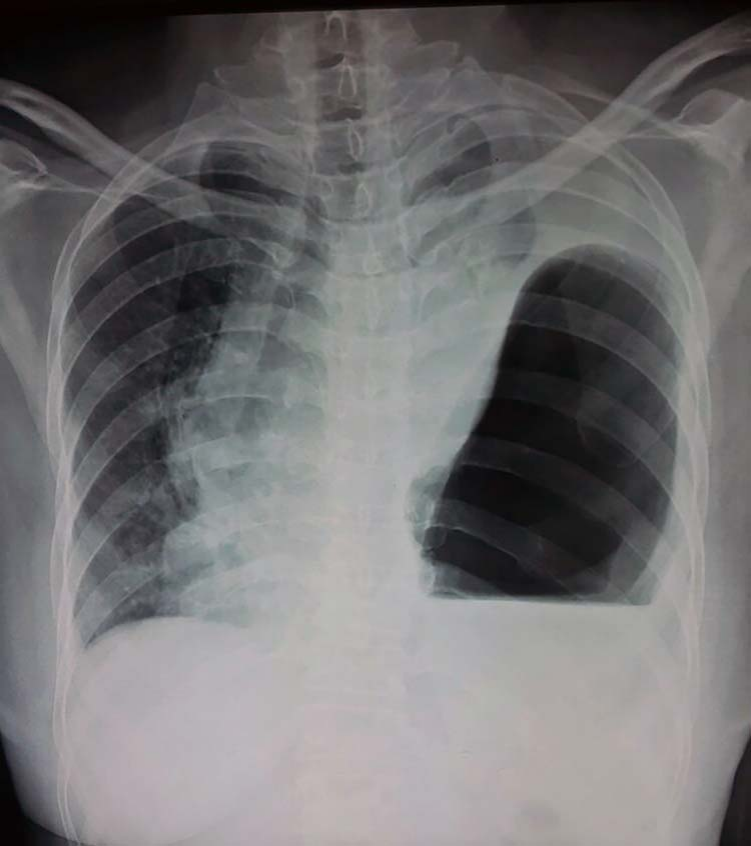
CXR showing left-sided air fluid level.

**Figure 2:**
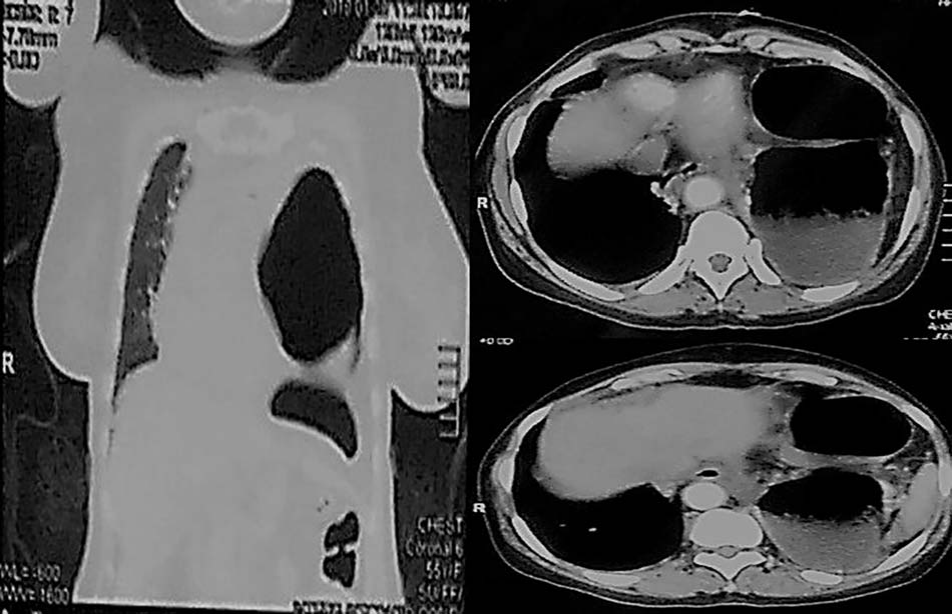
CT Thorax showing herniation of the stomach and spleen in the left chest cavity.

Her lab investigations showed total white blood cells count of 10.2109/L, heamoglobin 12.1 g/dl; renal and liver functions were within normal range. Her electrolytes were potassium 2.5 mmol/L, sodium 132 mmol/L and her Electrocardiography showed sinus tachycardia. Her initial chest X-ray (CXR) showed a left-sided air-fluid level with a circumferential opacity and mediastinal shift to the right side; there was an initial impression of tension pneumothorax (Fig. 1). But given the patient’s hemodynamic stability and radiologist advice, a computed tomography (CT) thoracic scan followed that showed a large left diaphragmatic hernia with the stomach and the spleen inside the left chest cavity causing significant mass effect with the result of total lung collapse (Fig. 2).

## DIFFERENTIAL DIAGNOSIS

The patient initially exhibited acute gastrointestinal symptoms pointing to an acute abdomen because of abdominal pain, vomiting and distension. The presentation to the hospital was associated with respiratory distress symptoms and signs on examination which could have led to a clinical misdiagnosis of tension pneumothorax at the early stages, but the patient’s hemodynamic stability allowed performing the CXR. The follow-up CT scan advised by the radiologist confirmed the diagnosis of an obstructed diaphragmatic hernia which would explain both the respiratory and gastrointestinal presentation.

## TREATMENT

**Figure 3:**
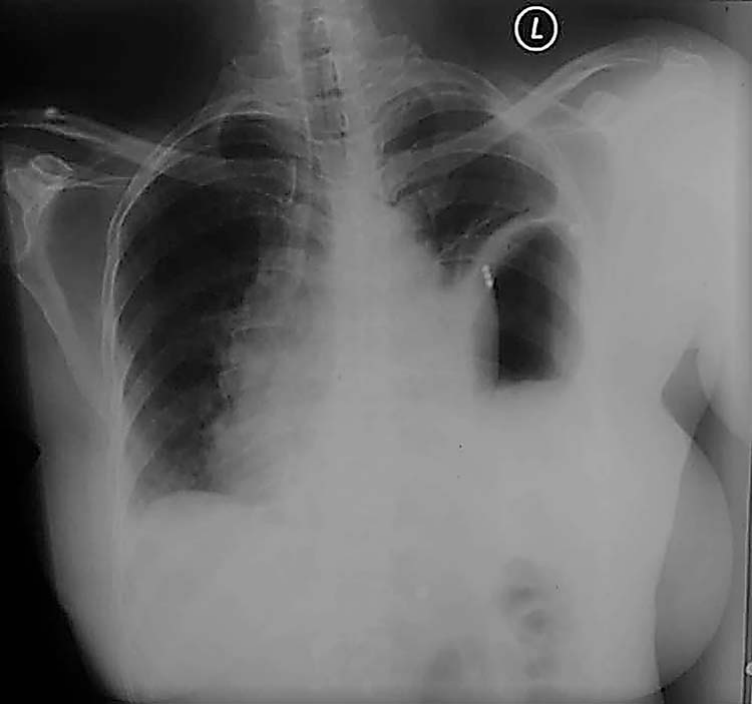
CXR Post nasogastric tube suction with marked left lung re-expansion.

**Figure 4:**
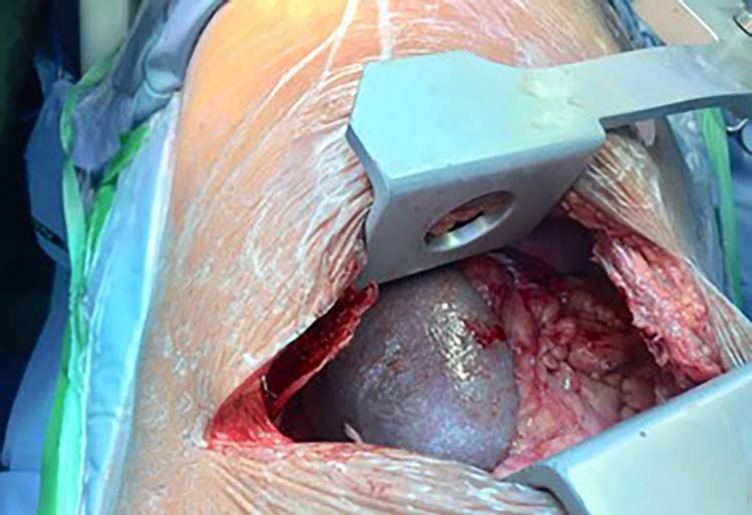
Left posterolateral thoracotomy revealing herniated spleen, stomach and greater omentum.

Following the CT scan and confirmation of diagnosis, a nasogastric tube was inserted. This drained 1.5 L of fluid over 2 h with a large quantity of air. A considerable improvement in the patient’s respiratory distress was noted with the respiratory rate normalizing and a follow-up CXR showed a dramatic reduction in the size of the intrathoracic stomach with a matching lung re-expansion (Fig. 3). She was brought to the operating room the following morning after appropriate resuscitation and electrolyte correction. The chest was entered via a seventh intercostal space leftposterolateral thoracotomy revealing a 5-cm defect in the left dome of the diaphragm with an incomplete hernia sac with the stomach, greater omentum and spleen incarcerated inside the chest cavity (Fig. 4). Adhesions were released and the stomach, omentum and spleen were reduced into the abdominal cavity. The defect was primarily closed in two layers (double breasting). The patient was sent home on the fifth post-op day following an uneventful recovery. She was seen 6 weeks later and was doing well.

## DISCUSSION

In their systematic review, Brown *et al*. [2] showed that 78% of symptomatic BHs occur on the left side similar to our case. A precipitating factor such as pregnancy, chronic constipation and rigorous physical activity can be found in 25% of symptomatic patients, none were found in our case [2]. Half of the patients present acutely with symptoms such as pain, pressure sensation in the abdomen or the chest, and symptoms of obstruction or pulmonary symptoms [2]. As these symptoms are ubiquitous, additional clinical clues would include postprandial respiratory symptoms, abdominal or thoracic symptoms aggravated by the supine position and abdominal symptoms aggravated by physical effort [3]. The results of the physical exam in our case gave rise to a diagnosis of pneumothorax. This highlights the importance of a careful analysis of the patient’s symptoms. The most common radiological modality used to diagnose CDH is the CXR followed by the CT scan, which is the most accurate modality [4]. Nasogastric tube decompression is a common initial management step in obstructed Hiatal hernias [5]. Using the same approach here improved the patient’s symptoms and allowed the surgical procedure to be performed in more of a semi-elective setting. It is proposed, though rarely, that rupture of the hernial sac may be the initiator of the progression towards a symptomatic hernia. That could have been the case with our patient as a herniated incomplete sac was found. It is recommended to repair any diaphragmatic hernias discovered, irrespective of the symptoms. We used primary repair in our case. Brown *et al*. [2] found that primary repair was the most performed with no reported recurrence.

## CONCLUSIONS

Obstructed Bochdalek hernias rarely exhibits symptoms in adults and should be suspected whenever gastrointestinal and respiratory symptoms intertwine to avoid potentially disastrous consequences.

## CONFLICT OF INTEREST STATEMENT

None declared.

## CONSENT

Informed consent has been obtained.
